# Treatment of Metformin-Containing Wastewater by a Hybrid Vertical Anaerobic Biofilm-Reactor (HyVAB)

**DOI:** 10.3390/ijerph16214125

**Published:** 2019-10-25

**Authors:** Eshetu Janka, Diego Carvajal, Shuai Wang, Rune Bakke, Carlos Dinamarca

**Affiliations:** 1 Department of Process, Energy and Environmental Technology, University of South-Eastern Norway, 3918 Porsgrunn, Norway; eshetu.j.wakjera@usn.no (E.J.); dcarvajal89@gmail.com (D.C.); rune.bakke@usn.no (R.B.); 2 Biowater Technology AS, Rambergvn 1, 3115 Tønsberg, Norway; sw@biowatertechnology.com

**Keywords:** integrated biological system, metformin, biofilm, anaerobic digestion, CFIC

## Abstract

Several series of batch and continuous experiments were performed to investigate the removal of metformin and other contaminants from two wastewaters: wastewater I (WWI) containing 4 mg/L metformin and wastewater II (WWII) containing 110 g/L butanol. Biomethane potential (BMP) tests on WWII showed 77% of total chemical oxygen demand (tCOD = 110 g/L) degradability, and no apparent inhibition effects were observed. BMP tests on WWI showed an apparent inhibitory effect reflected in lower biogas production with increasing metformin concentration in the wastewater. Continuous flow hybrid vertical anaerobic biofilm (HyVAB^®^) experiments were consistent with the batch test findings. It was necessary to co-digest WWI (metformin) with WWII (easily degradable organics) to achieve complete metformin removal. After a period of adaptation, WWI and WWII co-digestion achieved up to 98% tCOD removal and 100% metformin removal. Most of the contaminants were removed in the anaerobic section of the HyVAB^®^, which implies that higher chemical oxygen demand (COD) loads than tested here are possible, given some optimization. The pilot reactor was able to manage organic loads of 11 g COD/d and above 10 mg/L metformin with a removal of 98% and 100% for tCOD and metformin, respectively.

## 1. Introduction

The rapid development of large-scale pharmaceutical manufacturing has contributed to increased concentrations of contaminants present in wastewater and surface waters [1,2]. Pharmaceutical wastewaters are produced during the drug manufacturing processes (i.e., fermentation, extraction, chemical synthesis, formulation, packaging and washing of solid cake and equipment) [3,4]. Hence, the main pollution source of these pharmaceutical compounds in the environment is through industrial process water discharge [1,5]. Several studies have shown that pharmaceutical drugs (and their derived metabolites) are found in effluents of wastewater treatment plants (WWTPs) and their removal is very inefficient [1,6]. Wastewater originating during the manufacturing of antidiabetic drugs is also an important environmental challenge due to the high production volume [7,8]. Several research papers have reported the occurrence, degradability, and environmental impact of antidiabetic drugs in wastewater [9,10,11,12], and substantial efforts to achieve environmental improvements in line with regulations and legislation have been implemented [13].

Metformin (C_4_H_11_N_5_) is one of the most prescribed and widely used antidiabetic drugs worldwide, since a significant portion of the population suffers from diabetes [10,11,12]. Recent reports have indicated that there are also high concentrations of metformin in domestic wastewater treatment plants’ influent [12]. This is because it is excreted un-metabolized from the human body in urine and ends up in wastewater and aquatic environments [14]. Metformin is the pharmaceutical compound with the highest concentration in domestic wastewater (i.e., 64–98 µg/L) [15]. This study is therefore relevant for wastewater treatment in general, even though it is focused on metformin-containing industrial wastewater. Pharmaceutical wastewater has traditionally been treated using conventional physico-chemical and biological process methods [2]. Metformin can be aerobically degraded to guanylurea (C_2_H_6_N_4_O)—a metabolite that is stable against further biodegradation in wastewater treatment plants [9,11,12]. However, it appears to be a novel idea to integrate anaerobic and aerobic processes for the efficient removal of metformin and other pharmaceutical constituents. 

**Figure 1:**
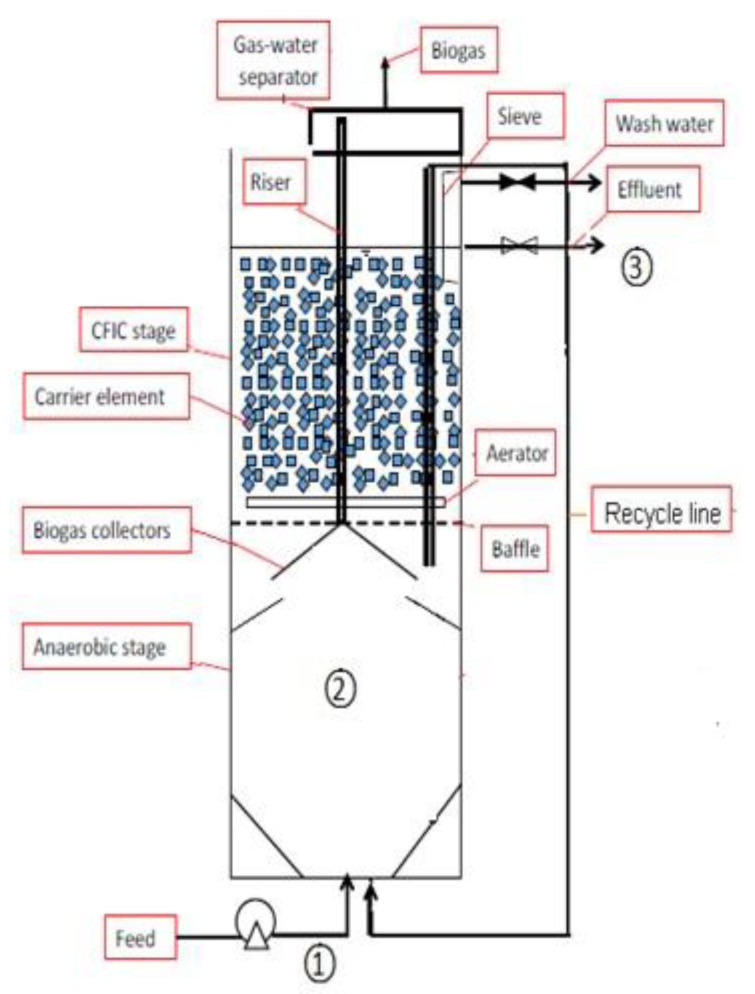
Hybrid vertical anaerobic biofilm (HyVAB^®^) bioreactor with anaerobic stage at the bottom and a Continuous Flow Intermittent Cleaning (CFIC^®^) stage at the top. (1) Influent; (2) Anaerobic section; and (3) Effluent, aerobic section [18].

The basic hypothesis of this study is that anaerobic granular sludge and aerobic biofilms supported by a hybrid vertical anaerobic biofilm (HyVAB^®^) reactor design (Figure 1) are the most robust combined niches for microorganisms to treat inhibitory substances, since microbial aggregates protect the microorganisms against such substances as they are delivered slowly by diffusion from the liquid phase. The more sensitive organisms are in the deeper layers protected by layers of organisms embedded in an extracellular polymeric matrix. Adaptation to toxic chemicals can be slow and can go through the stages of survival, tolerance, capture, and—if at all biodegradable—the final adaptation stage is degradation. The process can even remove non-biodegradable constituents by integration in the extracellular matrix to build more aggregates. 

HyVAB^®^ is a compact reactor that integrates both anaerobic and aerobic processes [16,17]. Wastewater is introduced at the bottom anaerobic digestion (AD) stage, where organic matter is degraded and converted into methane. The remaining unconsumed organics flow up for further treatment in the aerobic biofilm stage designed as a Continuous Flow Intermittent Cleaning (CFIC^®^). Plastic bio-carriers serve as a biofilm substratum in the aerobic biofilm stage, protecting the biofilm from shear force. Excess aerobic sludge is washed off during the intermittent washing process (i.e., CFIC^®^). Part of the detached aerobic biofilm settles down by gravity to the AD stage in the HyVAB^®^, where it is degraded simultaneously with the feed substrates [16,17]. In this study, two types of wastewater—wastewater I (WWI) and wastewater II (WWII)—were fed to the reactor, and performance was evaluated on the basis of metformin reduction, chemical oxygen demand (COD) removal efficiency, and biogas production. 

## 2. Materials and Methods 

### 2.1. Biomethane Potential and Biodegradability Batch Test

Methane potential tests were carried out using the AMPTS II (Automated Methane Potential Test System; Bioprocess Control^®^ (AMPTS II, Lund, Sweden) and the tests were performed according to the instructions for the AMPTS II given by the producer. Four different mixing ratios of WWI and WWII were tested in triplicates. The different mixing ratios resulted in different concentrations of metformin and COD load (Table 1). Granular sludge (100 mL) was used as inoculum for each treatment and the CH_4_ production of the inoculum (Control) was also determined in triplicate. The inoculum was pre-incubated at 35 °C for five days in order to deplete the residual biodegradable organic material present in it. 

The AMPTS II has 15 incubation bottles, where each bottle has 500 mL total volume (400 mL bulk liquid and 100 mL headspace). Each incubation bottle has a mechanical mixer rod that rotates at 60 rpm every 10 min. After filling the inoculum and the mixture feed in each bottle, the remaining volume was filled with distilled water to adjust the final working volume to 400 mL. Each bottle was tightly closed and flushed with N_2_ for 3 min to remove oxygen and create anaerobic conditions. The produced biogas from each incubation bottle went through a second bottle containing 3 M NaOH solution for fixing CO_2_, while CH_4_ passed through a gas counter. Gas was recorded via a digital pulse counter, and the output was normalized at standard temperature and pressure. 

### 2.2. Pilot-Scale HyVAB^®^ Design

The reactor (Figure 1) had a cross-sectional area of 0.016 m^2^ and height of 1.3 m with 13.5 L total volume—9 L for the anaerobic section and 4.5 L for the aerobic section. Between these sections there was a three-phase separator with three layers of baffles. The inlet feed was pumped from a mixing tank through the bottom of the anaerobic reactor using a digital pump (Master flex L/S, Vernon Hills, IL, USA). The biogas generated at the anaerobic section was collected by the three-phase separator and further channeled through a polyethylene tube to a volumetric gas meter.

The aerobic section contained highly packed biofilm carriers which filled >90% of the volume. The bio-carriers were made of high-density polyethylene and had a surface area of 650 m^2^/m^3^ with the dimensions of 14.5 × 18.5 × 7.3 mm^3^ (BWTS^®^, Biowater Technology AS, Biowater Technology AS, Tonsberg, Norway). To prevent the bio-carriers from falling down to the anaerobic section, a steel mesh was fixed above the three-phase separator. An air flow of about 400 L/h was supplied using an aeration tube fixed on the steel mesh. Air was regulated manually to obtain dissolved oxygen (DO) ≥ 3 ppm using a Dk 800 high-precision glass tube air flow meter. The DO was measured using a digital DO meter (WTW Oxi3310, Vernon Hills, IL, USA). 

The reactor was inoculated with anaerobic granular biomass obtained from a pilot-scale HyVAB^®^ reactor treating petrochemical wastewater. The granular sludge had a size range of 0.4–3.9 mm, a density of 1.1 kg/m^3^, and settling velocity of 138 m/h [17]. Aerobic biomass from the same pilot-scale reactor was used as inoculum for the aerobic section. The inoculum was exposed to metformin for several months before the study.

The reactor was operated in five phases (Table 2) with different feed composition and hydraulic retention time: (Phase I) Reactor start-up with synthetic feed to establish a strong culture. The synthetic feed was prepared using 1 g/L glucose, 0.5 g/L peptone, 3.5 g/L NaHCO_3_, 0.5 g/L K_2_HPO_4_, 0.4 g/L KH_2_PO_4_, 1 mL/L vitamins, and 1 mL/L mineral solutions prepared according to Dinamarca and Bakke [19]. The organic loading was 1.3 g COD/d, and the hydraulic retention time (HRT) was 9 days. (Phase II) The reactor was fed WWI with a metformin concentration of 4 mg/L (±0.5). The organic loading was 1.3 g COD/d, and the HRT was 2 days. (Phase III) The reactor was fed WWI and synthetic feed. (Phase IV) The reactor was fed WWI and WWII (most of the COD of WWII was butanol). WWII was also pharmaceutical wastewater produced during manufacturing processes. The mixing ratio between the two wastewaters was 1:2 (the same as the ratio available from the complete production process). The feed also contained 0.5 g/L peptone, 3.5 g/L NaHCO_3_, 1 mL/L of vitamins, 1 mL/L of minerals, 0.5 g/L KHPO_4_, and 0.4 g/L KH_2_PO_4_. The organic loading was 4.6 g COD/d with 7 days HRT, the reactor operated for 53 days. (Phase V) The reactor operated with similar feed as in the fourth phase except HRT was reduced to 4 days and the metformin concentration was increased to 5 mg/L by adding pure metformin as powder. The objective of phase V was to observe the reactor’s response to transient conditions such as a sudden increase in wastewater metformin concentration (increased 25%), since this may occur due to unintended disturbances in the metformin production. The reactor was then operated for 15 days. In total, the continuous experiment lasted 94 days. The reactor was operated at room temperature (22–23 °C). Wastewater compositions are presented in Table 3. 

### 2.3. Analytical Methods

Samples from influent, anaerobic section, and effluent (i.e., aerobic section) were taken two times per week for analysis. Total and soluble COD were measured by closed reflux colorimetric method, using the Merck Spectroquant^®^ (Darmstadt, Germany) COD cell test. The method corresponds to U.S. standard 5220 D [20]. To measure soluble COD, samples were centrifuged at 10,000 rpm for 30 min and then filtered with 0.45-μm pore size (GxF multi-layered, Acordisc^®^ PSF, Darmstadt, Germany) syringe filters before analysis. Total suspended solids (TSS), volatile suspended solids (VSS), and pH were also measured according to the U.S. standards 2540 D, 2540 E, 4500-H+ B [20], respectively. Determination of volatile fatty acids (i.e., acetate, propionate, butyrate, iso-butyrate, iso-valerate, and iso-caprionate) was conducted using gas chromatography (Hewlett Packard 6890, Santa Clara, CA, USA). The gas chromatograph had a flame ionization detector and a capillary column (FFAP 30 m, inner diameter 0.25 mm, film 0.5 μm). The carrier gas was helium at 23 mL/min. The oven was programmed to go from 100 °C at a rate of 15 °C/min, and then to 230 °C at a rate of 100 °C/min. The injector and detector temperatures were set to 200 °C and 250 °C, respectively. Biogas composition (CH_4_ and CO_2_) was analyzed using a gas chromatograph SRI-8610C. Metformin analysis was performed in HPLC according to Briones and Sarmah [21].

### 2.4. Data Analysis 

The means of each parameter measurement were used for statistical comparisons. Data analyses were done using R software (R version 3.3.3, www.r-project.org, Vienna, Austria) for outlier effect, normality test, equality, and analysis of variance (ANOVA). The removal of metformin with time at the aerobic condition was fit with the logistic model using R software (R version 3.3.3)

(1) $$C=\frac{\left(C_{0}+X_{0}\right)}{1+\left(\frac{X_{0}}{C_{0}}\right)e^{k\left(C_{0}+x_{0}\right)t}},$$

(2) $$k=\;\frac{{µ}_{max}}{K_{s}},$$

where *t* is time (day), *C*_0_ and *C* are the metformin concentrations (mg/L) at time *t*_0_ and *t*, respectively, *X*_0_ is the substrate required to produce the initial biomass concentration (mg/L), µ*_max_* is the maximum specific degradation rate (1/day), *K_S_* is the half-velocity constant (mg/L). The goodness of model fit was evaluated by comparing the sum of squared residuals (SSRes) and coefficient of determination (r^2^) [21].

## 3. Results and Discussions

### 3.1. Biomethane Potential Batch Test

**Figure 2:**
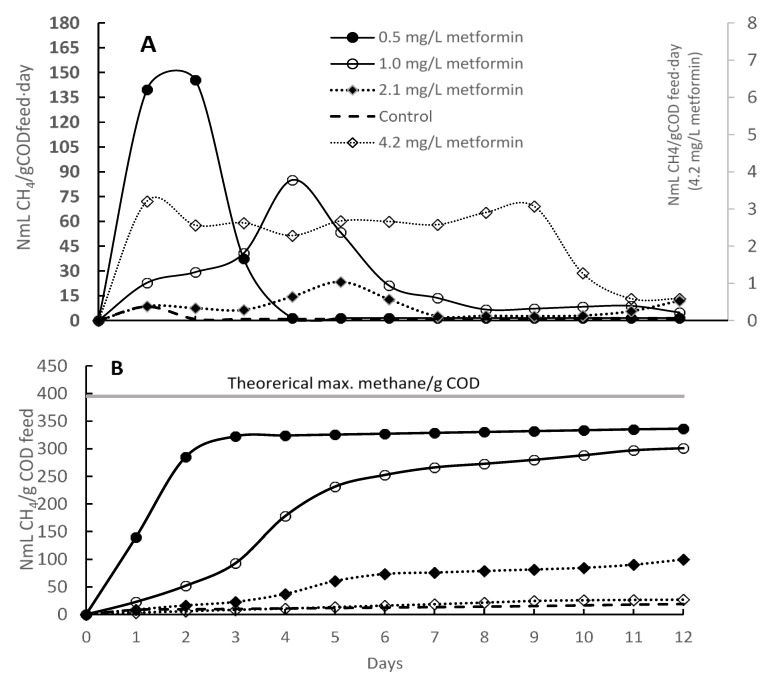
(**A**) Methane production rate; (**B**) Accumulated methane.

The methane potential test showed that biogas production rate and accumulation decreased with increased metformin concentration as shown in Figure 2A,B, respectively. The 0.5 mg/L concentration of metformin accumulated maximum biogas volume and reached maximum methane production within three days. Maximum production rates decreased by factors of 5, 19, and 56 when the concentration of metformin increased to 1.0, 2.1, and 4.2 mg/L, respectively (Figure 2A). The methane production for the feed containing 1 and 2 mg/L of metformin was delayed by two and three days, respectively (Figure 2B), compared to the 0.5 mg/L metformin concentration case. The accumulated methane production was lower by factors of 1.1, 3.4, and 12.5 for 1.0, 2.1, and 4.2 mg/L metformin compared to the 0.5 mg/L metformin concentration case, showing inhibition effect with increasing metformin concentration. The batch test with 0.5 mg/L metformin had a total biogas production equal to 85% of the maximum theoretical equivalent to COD. The low degradation of metformin may be due to the culture needing more adaptation time. Studies have confirmed that metformin can be degraded when a highly dense and diversified bacterial community is used. It is shown that differences in test results could be due to the diversity of the microbial community as well as to the presence of specific metformin-degrading bacteria [21,22,23]. 

### 3.2. Metformin Removal, HyVAB^®^ Continuous Experiment

During the first two weeks, the start-up period (phase I), the synthetic substrate enriched and enhanced biomass activity as observed by biogas production (data not shown). In phase II, where the reactor was fed only WWI containing 4 mg/L metformin, there was an initial 33% removal of the inlet metformin in the first week of operation and afterwards no metformin was removed at all, indicating inhibition of the biological activity. 

**Figure 3:**
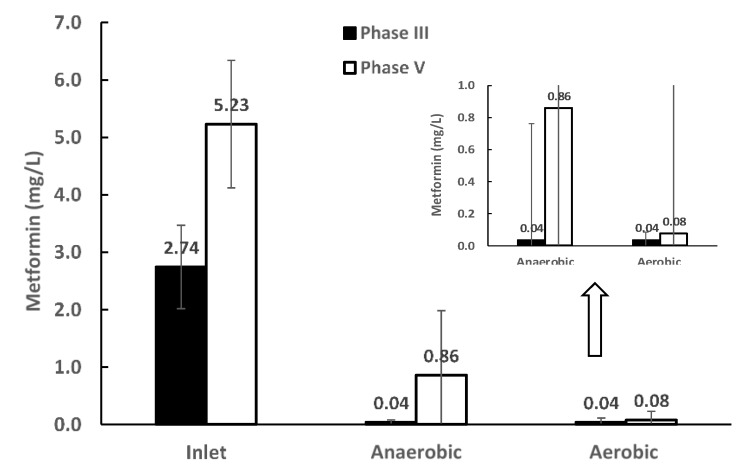
Metformin concentration in the inlet and effluents from the anaerobic and aerobic stages of the HyVAB^®^ in the experimental phases III and V. The error bars show the standard deviation (*n* = 3).

Biogas production recovered in the third phase of the experiment when metformin-containing wastewater was supplemented with a co-substrate (synthetic feed) to provide an easily degradable carbon source. Biomass activity recovered quickly and started to break down metformin, with almost no metformin residue measured in the reactor effluent (Figure 3). These results show that metformin and its intermediate (i.e., biotransformation product guanylurea) are biodegradable with an easily degradable carbon source co-substrate. Nearly complete removal of metformin was observed in the anaerobic section of the reactor, contradicting studies reporting that metformin is only aerobically biodegraded to guanylurea [11,22,24]. A recent study supporting our findings has indicated that complete degradation of guanylurea can also occur in the presence of easily degradable carbon sources [21].

Metformin (C_4_H_11_N_5_) consists of several nitrogen-rich amine groups, while guanylurea (C_2_H_6_N_4_O) contains fewer amine groups and has a lower carbon content. This comes to show that metformin is easily and completely degradable to guanylurea depending on the biomass activity and loading [21]. However, to facilitate the biodegradation of guanylurea, the supplementation of an easily degradable carbon source such as glucose is advantageous. Alternative carbon sources can also be used as substitutes for glucose since glucose is an expensive substrate. In our study, glucose was replaced by pharmaceutical process wastewater rich in butanol (WWII). 

Butanol is an important industrial chemical and is considered as a rich energy source [25]. When metformin (WWI) was co-digested with butanol (WWII) in the wastewater, metformin was degraded 84% in the anaerobic stage and reached 98% degradation after the aerobic stage (Figure 3). This shows that, with the supplement of easily degradable substrates, metformin and its biotransformation product guanylurea can be degraded almost (98%) completely. The biomass adapted to the new condition within two weeks. Hence, the quick response of the culture and high removal at the anaerobic section indicate that a higher metformin load than tested here could be handled by the bioreactor when co-digesting with an easily degradable organic substrate.

**Figure 4:**
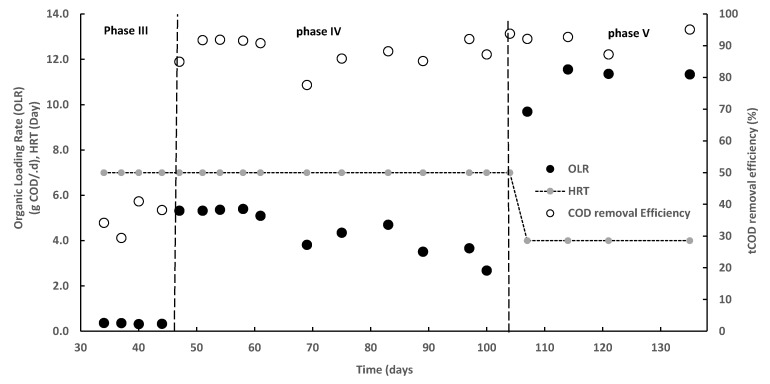
Total chemical oxygen demand (COD) removal efficiency of HyVAB^®^ at different organic loading rates (OLR) and hydraulic retention time (HRT) in the three operational phases when metformin was co-digested with organic source.

An active biomass was observed with the degradation of organics and biogas production. With sufficient carbon source the tCOD removal efficiency was on average close to 60% in phase III. The removal increased with increasing organic loading rate. In the phase IV, the average loading was 4.6 g COD/d, which is 72% higher than in the third phase, and the average tCOD removal increased to 94% (Figure 4). This indicates that an adapted culture can accommodate a higher metformin load when co-digesting with an easily degradable organic substrate. It was observed that biogas yield was in the range 86%–91% of influent COD after 40 days adaptation even when loading was increased and HRT reduced (Figure 4). Process adaptation to metformin was the focus in this study, while no effort was made to determine the maximum hydraulic and organic loading rates the process can handle. Therefore, HRT and OLR did not have an effect on tCOD removal in the range tested, since loading in this experiment was far below the reactor design capability 20–30 g COD/L·d [17] (Figure 4). This implies that a treatment plant operating at the HRT and OLR levels tested here has a significant safety factor, which may be appropriate given the challenging wastewater composition. HyVAB^®^ with its compact design has the advantage of a long sludge retention time at both the anaerobic (with granular sludge) and aerobic (with biofilm on carriers) sections, thus improving the removal rates of pollutants and allowing high loads. Organic material degraded in the anaerobic section produces methane that can be utilized. More difficult and slowly degradable compounds can be degraded at the aerobic section due to the higher redox potential. These features make HyVAB^®^ efficient in terms of energy demand, removal rates, and extent for the treatment of pharmaceutical wastewaters.

### 3.3. Degradation of Other Metabolites 

**Figure 5:**
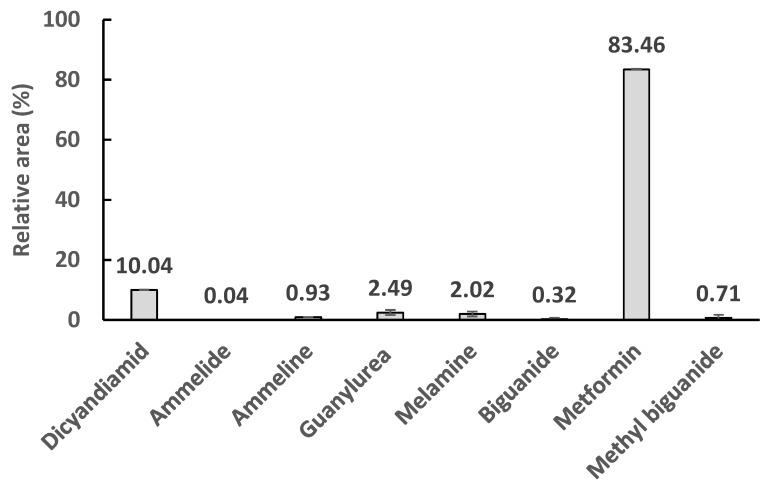
Measured metformin and other metabolites in the influent wastewater. The concentrations are expressed in relative areas of the chromatogram (i.e., each compound peak area is divided by the total area and expressed in percentage).

Metformin was biodegraded both aerobically and anaerobically to guanylurea, however during the hydrolysis and oxidation process there were several compounds identified at the inlet and outlet of the anaerobic and aerobic stages of the reactor (Figure 5). These were metformin-related compounds, for instance, biguanides are complex molecules which have been extensively studied for medicinal applications [26]. The most familiar cyanamide derivatives (e.g., melamine, ammeline, and ammelide) were also removed during the treatment. Ammeline is one of the sub-products in the hydrolysis of melamine which also converted to ammelide, but all have similar chemical reactivity [27].

Chromatograms show that several other compounds besides metformin were found in the wastewater, such as dicyandiamide and its derivatives melamine, ammelide, and ammeline. Their quantification was not possible due to a lack of appropriate standards, but with the exception of some dicyandiamide, almost all the observed hydrolysis derivatives were removed in the bioprocess. 

**Figure 6:**
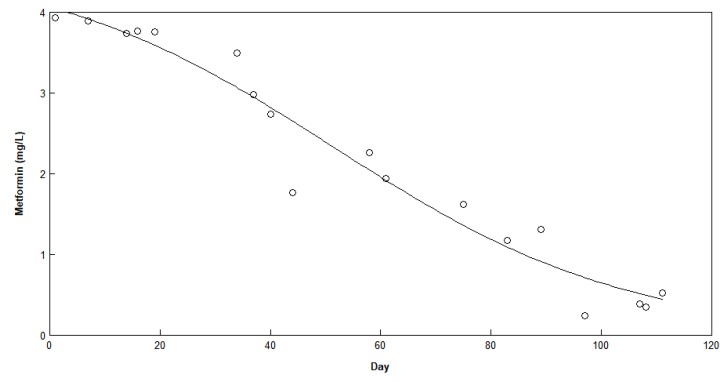
Metformin concentration over time without the addition of aerobic sludge. The logistic model is fitted on the data.

The degradation of metformin under aerobic conditions without adaptation was also observed through the continuous measurement of metformin over time. It was observed that even without any adapted sludge, metformin degraded slowly over time with a logistic model pattern (Figure 6). However, it took over 60 days to reach 50% metformin degradation, whereas with adapted culture in both anaerobic and aerobic stages the reactor removed close to 100% metformin and its associated compounds within 4 days.

## 4. Conclusions

Metformin present in wastewater could be completely degraded in the combined anaerobic and aerobic sections of the HyVAB^®^ reactor when an easily degradable organic co-substrate was present. Co-digesting metformin with an easily degradable substrate also enhances the degradation of metformin-related metabolites such as guanylurea. Slow and incomplete degradation in batch tests demonstrated the need for culture adaptation. Both anaerobic and aerobic microbes needed some adaptation period to metformin-containing wastewater to achieve efficient metformin removal. With the adapted culture, an increase in feed metformin concentration (25% to 5 ppm metformin) did not show any sign of inhibition, so adapted cultures could accommodate a higher metformin load than tested here, given an easily degradable organic co-substrate. Easily degradable organics could be available in other wastewaters from pharmaceutical manufacturing processes, such as the butanol-containing wastewater successfully tested here. Butanol proved to be a good organic co-substrate to remove metformin and its metabolites, such as guanylurea. 

## Figures and Tables

**Table 1:** Treatment combination and metformin content for the Automated Methane Potential Test System (AMPTS) test. COD: chemical oxygen demand; WWI: wastewater I; WWII: wastewater II.

Treatments (Triplicates)	Sludge (mL)	Feed WWI (mL)	Feed WII (mL)	Feed COD (g)	Metformin (mg/L)
1 (control)	100	-	-		
2	100	10	5	0.55	0.5
3	100	20	10	1.10	1.0
4	100	40	20	2.21	2.1
5	100	80	40	4.42	4.1

**Table 2:** Continuous operation parameters, inlet metformin concentration, hydraulic retention time (HRT) and organic loading rate (OLR).

Operation Phases	Feed	Days	Metformin (mg/L)	HRT (d)	OLR (g COD/d)
I	Synthetic feed	14	0	9	1.3
II	WWI	30	4	2	1.3
III	WWI + Synthetic feed	12	4	7	1.3
IV	WWI + WWII	53	4	7	4.6
V	WWI + WWII	15	5	4	11.4

**Table 3:** Wastewater parameters of relevance for biological treatment.

Wastewater Characteristics	WWI	WWII
Total COD (g/L)	0.23	110
Soluble COD (g/L)	0.14	107
Total solids (TS) (mg/L)	0.17	39.4
Volatile solids (VS) (mg/L)	0.16	16.3
Total suspended solids (TSS) (mg/L)	0.04	0.04
Volatile suspended solids (VSS) (mg/L)	0.04	0.01
pH	6.2	11.6
Alkalinity (mg CaCO_3_/L)	30	12500
NH_4_^+^-N (mg/L)	3.7	<4.0
